# A Systematic Review of Emergent Learning Outcomes Produced by Foreign language Tact Training

**DOI:** 10.1007/s40616-022-00170-z

**Published:** 2022-07-06

**Authors:** John R. Wooderson, Lewis A. Bizo, Kirsty Young

**Affiliations:** 1 University of Technology Sydney, Sydney, Australia; 2 Lojic Institute, Loganholme, Australia; 3 Present Address: The Kameleon Group, Sydney, Australia

**Keywords:** emergent learning, foreign language learning, second language learning, tact training

## Abstract

This systematic review evaluated the effects of foreign tact training on emergent learning outcomes in ten published studies. We also conducted a meta-analysis of aggregate data from seven studies comparing outcomes of foreign tact training with other verbal operant procedures. The preliminary findings indicated foreign tact training produced criterion-level responses in 84 of 106 (79.2%) post-test probes across 37 learners and 55 evaluations of foreign tact training. The meta-analysis results revealed significantly higher within-subjects mean levels of emergent responding following foreign tact training than foreign-to-native intraverbal, native-to-foreign intraverbal, and foreign listener training. Emergent outcomes for adults were not significantly greater than for children. Finally, foreign tact training was slightly more efficient than the other verbal operant procedures, although most of the differences were not statistically significant.

**Supplementary Information:**

The online version contains supplementary material available at 10.1007/s40616-022-00170-z.

Learning a foreign language is thought to provide a range of cognitive (Antoniou et al., 2013; Cheng et al., 2015), emotional (Gómez, 2016), and financial (New American Economy, 2017) benefits. Learning a foreign language may be costly and time-consuming, with some languages requiring at least 2200 hours (88 weeks) of study to develop fluent performance (U.S. Department of State, n.d.). Furthermore, some programs use considerable education resources. South Korea, for example, spent 40% (12 billion dollars) of its public education budget on English language programs in 2009, and private education costs were estimated to be even higher (Piller, 2016). In the European Union, up to 95% of students in upper secondary education study a foreign language (European Commission, 2020). Given the potential cost of foreign language study, educators must optimize learning by making instruction efficient. In this regard, behavior analysis has much to offer as the field’s history is replete with empirical demonstrations of evidence-based instructional procedures (Binder & Watkins, 1990; Vargas, 2020). This review examines foreign tact training (FTT)—a promising behavior-analytic procedure for efficient foreign language learning.

Traditional language theories view verbal operants, such as speaking and listening behaviors, as innately interdependent (e.g., Chomsky, 1957; Kuhl, 2004). However, various behavior-analytic accounts contend that these operants are initially independent but may become ‘joined’ through repeated incidental experiences, modeling, and direct reinforcement (Greer & Speckman, 2009). Furthermore, the learner’s integration of these capabilities represents a generalized verbal operant that allows for potentially unlimited patterns of emergent responding and generalized language development. Three main theories—stimulus equivalence (e.g., Sidman, 1971), naming theory (e.g., Horne & Lowe, 1996), and relational frame theory (RFT; e.g., Barnes-Holmes et al., 2018)—have been developed to understand the conditions that occasion derived stimulus relations and emergent learning (Critchfield et al., 2018; Lafrance & Tarbox, 2020; Rehfeldt, 2011). Sidman’s (1971) influential study on stimulus equivalence discovered that untrained relations could emerge following the teaching of certain stimulus-response relations. Relational frame theory further builds upon stimulus equivalence by conceptualizing equivalence and other stimulus relations as classes of generalized relational operants, which are referred to within RFT as relational frames. Engaging in relational responding is occasioned by contextual cues that function as discriminative stimuli for previously established patterns of relational responding (Barnes-Holmes et al., 2018). According to RFT proponents, learners develop relational frames due to a reinforcement history of relational exemplars. In naming theory (Horne & Lowe, 1996), naming refers to the learner’s combination of speaker and listener behaviors. These three theories have generated extensive research and a broad range of empirically validated language development and learning procedures.

A growing field of study has emerged in the behavior analytic literature examining the efficacy of behavior-analytic based procedures for foreign language learning. This literature applies emergent learning practices and verbal operants to foreign vocabulary training (Daly & Dounavi, 2020). The languages taught include Native American (Haegele et al., 2011), Japanese (Petursdottir et al., 2014), French (Daly & Dounavi, 2020; Polson et al., 1997; Polson & Parsons, 2000), German (Rocha e Silva & Ferster, 1966), Spanish (Joyce & Joyce, 1993; Matter et al., 2020; Petursdottir et al., 2008; Ramirez et al., 2009), Italian (Petursdottir & Hafliđadóttir, 2009), Chinese (Wu et al., 2019), Welsh (May et al., 2016, 2019), and English (Cortez et al., 2020, 2021; Dounavi, 2011, 2014; Rosales et al., 2011, 2012). This literature’s defining feature is its focus on emergent learning as a critical outcome of effective foreign language instruction.

Behavior analysts value emergent learning because it represents what might be characterized as “free” knowledge or skills that do not require direct experience (e.g., Critchfield et al., 2018; Critchfield & Twyman, 2014). However, if learning goals are limited to what may only be explicitly taught, then the scope and breadth of outcomes are also limited (Critchfield, 2018). Instead, the instructor expects untrained operants to emerge following a carefully selected subset of learning content (Critchfield, 2018; Dixon & Stanley, 2020). Studies in this field have implemented training procedures involving a range of verbal operants, including listener behavior (e.g., Rocha e Silva & Ferster, 1966), echoics (e.g., Petursdottir et al., 2014), mands (e.g., Wu et al., 2019), native-to-foreign intraverbals (NFI; e.g., Petursdottir & Hafliđadóttir, 2009), foreign-to-native intraverbals (FNI; e.g., Polson & Parsons, 2000), and tacts (e.g., Petursdottir et al., 2008) and tested for the emergence of untrained verbal operants.

Skinner (1957, p. 83) considered the tact the most important verbal operant because mands, intraverbals, and listener relations often depend on the learner’s ability to reference a wide range of environmental stimuli (Sundberg, 2015). Consequently, a strong tact repertoire is vital to social and academic success (Bak et al., 2021; Lalonde et al., 2020). Foreign tact training involves teaching learners to tact environmental stimuli using appropriate foreign language referents. Following FTT, learners may acquire several untrained relations, including listener responses, intraverbals, and mands in addition to the trained tacts. Among the various teaching procedures, FTT may be the most productive; several studies have noted its superior efficiency (e.g., Cortez et al., 2020, 2021; Daly & Dounavi, 2020; Dounavi, 2011; Matter et al., 2020). In emergent learning, efficiency means the amount of and ease with which learners acquire the trained and untrained material (Dounavi, 2011).

Recently, Matter et al. (2020) showed that FTT alone was more efficient than a traditional multi-component procedure comprising four verbal operants (tact, FNI, NFI, and listener training). Using an adapted alternating treatment design, the authors provided Spanish-language training to four English-speaking children. The results showed FTT required fewer sessions to mastery than the multi-component procedure and resulted in almost all learners acquiring emergent receptive and productive relations despite not receiving any training in the FNI, NFI, and listener relations. In addition, FTT produced more efficient emergent FNI and NFI responses than listener training with Portuguese-speaking Brazilian children learning English in studies by Cortez et al. (2020, 2021). However, the authors noted FNI and NFI relations did not always emerge at comparable levels. Dounavi (2011, 2014) conducted two methodologically similar studies with adult native-Spanish speakers. Both studies compared FTT with FNI training and NFI training. In the earlier study (Dounavi, 2011), FTT achieved higher levels of emergent responding and required fewer training trials than FNI or NFI training for both participants. In Dounavi (2014), on the other hand, NFI relations took fewer trials to achieve mastery criterion than the foreign tact relations; so NFI training was the most efficient condition. When Daly and Dounavi (2020) systematically replicated and extended Dounavi (2014), they used a modified concurrent multiple probe design to improve internal validity. Their results were comparable with Dounavi (2014); FTT produced more emergent responses than FNI or NFI training. However, FTT needed fewer trials to criterion. Furthermore, probes at four weeks post-training showed better maintenance of emergent responses following FTT than the two intraverbal conditions.

Foreign tact training is not successful for all learners, though. For example, Wu et al. (2019) compared the effects of FTT, FNI training, NFI training, and mand training in Mandarin Chinese vocabulary. They found FTT was the most efficient procedure for only one of the four participants. Also, May et al. (2019) reported equivocal results—robust increases in derived intraverbal relations after FTT for only half of the children in their study. Some researchers (e.g., Daly & Dounavi, 2020; Dounavi, 2014; Petursdottir & Hafliđadóttir, 2009) suggest young children are less likely to produce emergent responses because they are less verbally competent than adults. However, we could find no studies directly comparing emergent foreign language learning outcomes between adults and children.

In summary, emergent learning and FTT offer considerable potential for optimizing foreign language programs. However, it is difficult to determine the generality of FTT outcomes as the available research is limited to single-case experimental studies. Thus, it is unclear whether FTT is more efficient than other verbal operant training procedures at the group level analysis. This paper aimed to extract and analyze aggregate data from the literature on FTT use. In doing this, we considered the following three questions. First, what are the effects of FTT on emergent learning outcomes in the published literature to date? Second, how do FTT acquisition, emergence, and overall efficiency compare with other verbal operant training procedures? Finally, does FTT produce higher levels of emergent responding for adults or children?

## Method

### Literature Search Procedure

The search included APA PsycINFO (EBSCOhost), Medline (EBSCOhost), ERIC (EBSCOhost), CINAHL (EBSCOhost), APA PsycArticles (EBSCOhost), Psychology and Behavioral Sciences Collection (EBSCOhost), SocINDEX (EBSCOhost), and Web of Science electronic databases for English language studies published in peer-reviewed journals, with no limit specified regarding the year of publication. In addition, we combined various keyword terms related to emergent learning (emerg*, derive*, equivalenc*, generative*), foreign language learning (foreign language, second language), and verbal operant training (mand, tact, intraverbal, echoic, textual, dictation, autoclitic, verbal behavi*, verbal operant, match-to-sample, conditional discrimination, multiple exemplar). Finally, the wildcard * expanded the search to include all variants of the keywords.

**Fig. 1:**
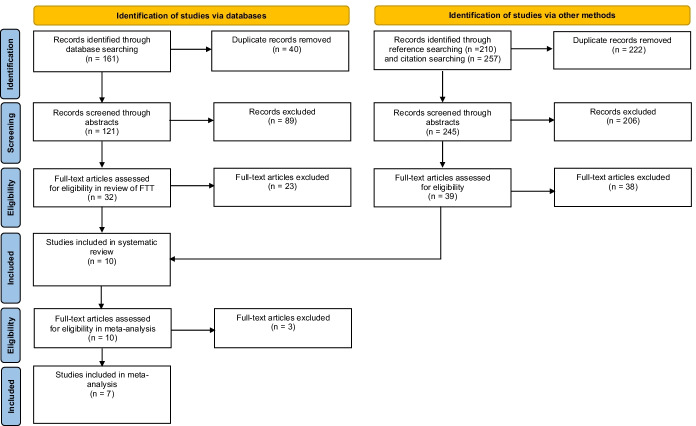
PRISMA chart showing systematic literature search sequence (Page et al., 2021)

The search sequence (Fig. 1) initially identified 161 articles—121 after removing duplicates. The first author then reviewed the abstracts of all 121 unique articles and removed all non-English articles, non-empirical papers (review, policy, position, commentary, or conceptual articles), and studies focused on language use (linguistic, diagnostic, textual, historical, cultural, psychometric, phonological, content, orthographic, or discourse analyses). We screened the remaining 32 full-text articles for three inclusion criteria: The experimenters focused on observable and measurable foreign language targets; the experiment included at least one standalone FTT procedure; the procedures involved at least one pre- and post-test for untrained emergent relations. We excluded studies with native-, contrived-, artificial-, nonsense-, or non-language learning targets and studies that combined FTT with other verbal operant procedures. We allowed, however, studies with native-tact pre-training trials—checks to see whether learners could tact the stimuli in their native language.

After initial full-text eligibility screening, we identified nine articles that met the criteria (Cortez et al., 2020, 2021; Daly & Dounavi, 2020; Dounavi, 2014; Matter et al., 2020; May et al., 2019; Petursdottir & Hafliđadóttir, 2009; Petursdottir et al., 2008; Wu et al., 2019). We then conducted reference and citation searches using Google Scholar and Web of Science. These searches returned a further 245 potential papers, which we also assessed for eligibility—yielding one additional article (Dounavi, 2011). In total, ten articles were included that contained 55 distinct evaluations of FTT. We excluded Matter et al.’s (2020) ‘mixed’ training evaluation from our sample because these trials combined FTT, NFI, FNI, and listener training. Furthermore, “mixed” training post-tests in Matter et al. (2020) evaluated directly trained relations only.

We also evaluated a subset (seven) of the ten FTT studies through meta-analysis (Cortez et al., 2020, 2021; Daly & Dounavi, 2020; Dounavi, 2011, 2014; Petursdottir & Hafliđadóttir, 2009; Wu et al., 2019). We only included studies in the meta-analysis if they contained at least one within-subject evaluation comparing the emergent learning outcomes produced by FTT with at least one other verbal operant training condition. Consequently, we excluded Petursdottir et al. (2008) from the meta-analysis because it did not contain any within-subject evaluations of training conditions. In addition, we excluded Matter et al. (2020) and May et al. (2019) because neither study compared the emergent learning outcomes following FTT with those produced by other verbal operant training procedures. As noted above, Matter et al. (2020) taught all target relations in the “mixed” training condition directly, meaning they could only test for emergent relations following FTT; May et al. (2019) implemented FTT only.

### Data Categorization

The ten articles were categorized according to participant demographic data (age, gender, native language, setting), target foreign language, types of training conditions employed, and mastery criteria for instructional and emergent learning outcomes. Each of the 55 FTT evaluations was coded according to whether it produced criterion-level responses in post-training probes. All experiments probed two or more distinct types of emergent relations; we evaluated each relation separately, where appropriate. The post-test results for each emergent relation were categorized as either achieving or not achieving criterion levels. If studies stated no specific mastery criteria, we set a criterion of 100%. Some evaluations included more than one post-test probe per emergent relation—we only included the highest post-test score recorded for each relation.

We evaluated the quality of each study using criteria as recommended by Schlosser and Sigafoos (2007): experimental design; follow-up data collected after three months, at minimum, for at least 90% of the participants; appropriate and independently assessed reliability measures; and counterbalancing or random allocation of stimuli to training conditions. We also evaluated the studies against the Council for Exceptional Children (CEC, 2014) quality standards for evidence-based practices. The CEC standards include 22 indicators for assessing the quality of single-case experimental studies, which can be used to determine whether an instructional procedure qualifies as an evidence-based practice.

### Data Extraction for Meta-analysis

The meta-analysis evaluated training acquisition rates, emergent post-test scores, and the overall efficiency of each verbal operant training procedure. The first author extracted data from the seven papers’ graphs and tables using DigitizeIt (Bormann, 2020). Concurrently, we emailed the corresponding author of each study once and requested the training and post-test data to conduct our analyses. We received written responses from six authors—one of whom stated they had not retained the data, and another noted the data were not immediately available. We did not receive a response from one author. Our requests resulted in raw data for four papers (Cortez et al., 2020; Daly & Dounavi, 2020; Dounavi, 2011, 2014). We did not send any follow-up requests; rather, we utilized the software-extracted data only for the remaining three papers.

Following data extraction, we regraphed the acquisition curves from each study on standardized panels and compared acquisition rates using descriptive visual analysis methods. Then, we calculated standardized acquisition rates (SAR), which represent the average number of training trials needed per word learned. To calculate SARs, we multiplied the number of trial blocks by the number of trials per block and divided by the number of items trained and the terminal percent correct; SAR = (number of trial blocks *number of trials per block) / (number of items per training set) / (terminal % correct) *100). The smaller the resulting value, the better the SAR. By including “terminal % correct” in the calculation, we could weight scores and compare training evaluations with different mastery criteria. Furthermore, we could compare training evaluations that researchers discontinued before the learner reached the mastery criterion.

We then compared FTT emergent post-test results with FNI, NFI, listener, and mand training post-tests. We did this by converting all post-test scores to percentages and calculating mean scores for each training evaluation within and across each study. Then, we conducted within-subjects statistical analyses using mean post-test scores for each learner and each training condition in which they participated. The analyses comprised Wilcoxon signed-rank non-parametric dependent-samples tests conducted in Jamovi (The jamovi project, 2020). We included all post-tests for emergent tact, FNI, NFI, and mand relations but excluded all tests for emergent listener relations (six scores) from the analysis due to the potential confounds of comparing unbounded scores with scores bounded by chance (Petursdottir & Hafliđadóttir, 2009). Three studies implemented reverse intraverbal training with participants following initial post-test probes (Daly & Dounavi, 2020; Dounavi, 2011, 2014). Consequently, we only included post-test data from the initial training sequence to control confounds associated with potential sequencing effects. We also used a Mann–Whitney U non-parametric independent-samples test to compare children’s mean FTT post-test scores (under 18 years) and adults (18 years and older). Lastly, we evaluated the overall efficiency of each verbal operant procedure by calculating an efficiency index score (EIS) using the SAR and mean post-test scores described above; EIS = mean post-test / SAR. The larger the resulting value, the better the EIS. We then analyzed the EIS data using Wilcoxon signed-rank non-parametric dependent-samples tests.

### Interobserver Agreement

The first author and an independent rater (BCBA-D®) read the full text of 31 articles and evaluated their eligibility based on the inclusion and exclusion criteria. The mean agreement was 100%. The first author also compared the data from four articles (Cortez et al., 2020; Daly & Dounavi, 2020; Dounavi, 2011, 2014), extracted using DigitizeIt, to the raw data provided by the authors. In total, we evaluated 88 (57.1%) post-test scores and 688 (80.0%) training trial scores. The mean agreement was 100%.

## Results

### Participant Demographics

Table 1 summarizes the demographic data, types of emergent relations tested, and mastery criteria (if any) stated by the authors. Across the 10 studies, 26 participants were children, and 11 were adults. Eight studies reported data on individual participant age; the mean participant age across 27 children and adults was 15.8 years (range 4–40 years). The mean age of children (n = 16) was 5.0 years (range 4–6 years), and adults (n = 11) was 31.5 years (range 23–40 years). The remaining two studies reported the range of participants’ ages only (n = 10; range 7–9 years). Just five studies directly reported participant gender, including six females and nine males. Participants’ native language was reported as English (n = 17), Portuguese (n = 10), Spanish (n = 4), or Icelandic (n = 6). The studies occurred in various settings, with the highest number (n = 4) conducted in learners’ homes.

**Table 1:** Data extraction of studies included in the systematic review

Paper	Participants	Setting and target foreign language	Instructional conditions	Types of emergent relations probed post-FTT	Instructional mastery criteria	Mastery criteria for emergent post-test probes	Number of criterion level emergent relations for FTT	Number of non-criterion level emergent relations for FTT
Cortez et al. (2020)	Six Portuguese-speaking children. Age range 7–9 years	Lab room English	FTT, listener behavior training	FNI, NFI	100% correct responses in three consecutive trial blocks	None stated	11* (5 NFI, 6 FNI)	1* (NFI)
Cortez et al. (2021)	Four Portuguese-speaking children. Age range 7–9 years	Lab room English	FTT, listener behavior training	FNI, NFI	100% correct responses in three consecutive trial blocks	None stated	7* (4 NFI, 3 FNI)	1* (FNI)
Daly and Dounavi (2020)	Three English speaking adults – 31M, 33F and 40F	Home French	FTT, intraverbal training (FNI, NFI)	FNI, NFI	100% correct responses in two consecutive trial blocks	10 out of 10 correct responses in one probe. A second probe session was conducted if a participant scored less than 10 but at least 7	6 (3 NFI, 3 FNI)	0
Dounavi (2011)	Two adult native Spanish speakers – 36M and 39M	Home English	FTT, intraverbal training (FNI, NFI)	FNI, NFI	100% correct responses in two consecutive trial blocks	30 out of 30 correct responses in one probe A second probe session was conducted if a participant scored less than 30 but at least 27	8 (4 NFI, 4 FNI)	0
Dounavi (2014)	Two adult native Spanish speakers – 37M and 29F	Home English	FTT, intraverbal training (FNI, NFI)	FNI, NFI	100% correct responses in two consecutive trial blocks	30 out of 30 correct responses in one probe A second probe session was conducted if a participant scored less than 30 but at least 27	8 (4 NFI, 4 FNI)	0
Matter et al. (2020)	Four 4-year-old English speaking children	School Spanish	FTT, mixed training (FTT, FNI, NFI, listener behavior)	Listener, FNI, NFI (only following FTT)	83.3% correct responses for two consecutive trial blocks	10 out of 12 correct responses in one probe	16 (4 NFI, 5 FNI, 7 Listener)	5 (3 NFI, 2 FNI)
May et al. (2019)	Six English speaking children (two 5-year-olds, four 6-year-olds)	School Welsh	FTT (group choral) only	FNI+NFI (mixed intraverbal trials)	89% correct responses for one trial block	None stated	10* (mixed intraverbals - NFI+FNI)	7* (7 mixed intraverbals - NFI+FNI)
Petursdottir and Hafliđadóttir (2009)	Two 5-year-old native-Icelandic speakers	Pre-school Italian	FTT, intraverbal training (FNI, NFI), listener behavior training	Listener, FNI, NFI	83.3% correct responses for two consecutive trial blocks	10 out of 12 correct responses in one probe	3 (1 NFI, 2 Listener)	3 (1 NFI, 2 FNI)
Petursdottir et al. (2008)	Four 5-year-old native-Icelandic speakers	Pre-school Spanish	FTT, listener	FNI, NFI	100% correct responses in three consecutive trial blocks	None stated	7* (4 NFI, 3 FNI)	1* (FNI)
Wu et al. (2019)	Four native English-speaking adults 26M, 26M, 26M, 23F	University-based clinic and home Mandarin Chinese	FTT, intraverbal training (FNI, NFI), mand training	Mand, NFI, FNI	83.3% correct responses for two consecutive trial blocks	None stated	8* (2 NFI, 4 FNI, 2 Mand)	4* (2 NFI, 2 Mand)

*FTT*, foreign tact training, *NFI*, native-to-foreign intraverbal, *FNI,* foreign-to-native intraverbal, *M*, male, *F*, female.

*Denotes studies that did not state mastery criteria for emergent relations—for which, we set a criterion of 100%

### Target Foreign Languages, Training Conditions, Mastery Criteria, and Emergent Learning Relations

The ten studies targeted six foreign languages—four trained English vocabulary to non-English speaking learners (Cortez et al., 2020, 2021; Dounavi, 2011, 2014). Other than the one study that focused on Mandarin Chinese words (Wu et al., 2019), all target foreign languages were European: English (n = 4), Spanish (n = 2), French (n = 1), Italian (n = 1), and Welsh (n = 1).

In addition to FTT, studies included a range of verbal operant training procedures: FNI, NFI, listener behavior, and mands training. Instructional mastery criteria ranged from 83.3% correct responses across two consecutive sessions (Matter et al., 2020; Petursdottir & Hafliđadóttir, 2009; Wu et al., 2019) to 100% correct responses across three consecutive sessions (Cortez et al., 2020, 2021; Petursdottir et al., 2008). Additionally, FTT studies tested a range of untrained relations: FNI, NFI, listener, and mands. Notably, all ten studies tested for emergent intraverbal (FNI and NFI) relations post FTT. Although only five studies stated specific mastery criteria for emergent relations, the reported standards varied from 83.3% (Matter et al., 2020; Petursdottir & Hafliđadóttir, 2009) to 100% correct (Daly & Dounavi, 2020; Dounavi, 2011, 2014). Five studies did not specify any mastery criteria for emergent relations; in which case, we set a conservative criterion of 100% correct (Cortez et al., 2020, 2021; May et al., 2019; Petursdottir et al., 2008; Wu et al., 2019).

### FTT’s Emergent Learning Outcomes

Table 1 also shows FTT’s emergent learning outcomes in each of the ten studies (55 FTT evaluations). In total, 84 (79.2%) post-test probes scored at or above criterion level responding and 22 (20.8%) scored below. Overall, FNI relations (84.2%) emerged at mastery criterion levels slightly more often than NFI (81.6%). Furthermore, FTT produced criterion-level emergent listener relations for all six learners in the two studies with listener probes (Matter et al., 2020; Petursdottir & Hafliđadóttir, 2009). However, chance-level responding for listener probes was 33% (Petursdottir & Hafliđadóttir, 2009). In contrast, FTT produced criterion-level mand relations in only 50% of probes, although only one study included tests for emergent foreign mands (Wu et al., 2019).

Table 2 shows that the studies achieved most of the quality standards recommended by Schlosser and Sigafoos (2007), except for follow-up data and experimental design. For example, although Matter et al. (2020) included long-term follow-up data beyond three months post-training, they only conducted sessions with two of the four participants. Additionally, most studies employed robust experimental designs to evaluate the effects of training procedures on the trained relations, but only five studies used control conditions or multiple-baseline designs when evaluating emergent relations (Matter et al., 2020; May et al., 2019; Petursdottir & Hafliđadóttir, 2009; Petursdottir et al., 2008; Wu et al., 2019). Three of the ten studies met all 22 CEC quality indicators (Matter et al., 2020; May et al., 2019; Wu et al., 2019). Most studies that did not meet all 22 quality indicators failed to include an evaluation of treatment integrity (Cortez et al., 2020; Daly & Dounavi, 2020; Dounavi, 2011, 2014; Petursdottir & Hafliđadóttir, 2009; Petursdottir et al., 2008). Other reasons studies fell short of the CEC standards included not having at least three data points in post-test phases or robust controls for threats to internal validity (e.g., control conditions or multiple-baseline designs). Based on these results and the CEC (2014) standards, the review’s findings indicate FTT is a potentially evidence-based practice.

**Table 2:** Analysis summary of key research paper methodological design and analytic elements

Paper	Experimental design (trained relations)	Experimental design (emergent relations)	Follow-up data	Reliability measures	Counterbalancing or random allocation of stimuli to conditions	CEC standards outcomes (total 22)
Cortez et al. (2020)	AATD	Pre- post-test probes	No	Yes	No	17
Cortez et al. (2021)	AATD	Pre- post-test probes	Yes, at two weeks or one month for two of the four participants	Yes	Yes	20
Daly and Dounavi (2020)	Modified MBL probe	Pre- post-test probes	Yes, at four weeks post-training	Yes	Yes	18
Dounavi (2011)	MBL across participants and stimulus sets	Pre- post-test probes	No	Yes	No	16
Dounavi (2014)	MBL across participants and stimulus sets	Pre- post-test probes	No	Yes	Yes	16
Matter et al. (2020)	AATD embedded within MBL and a control condition	Pre- post-test probes with control conditions	Yes, at 2- and 4-months post-training for two of the four participants	Yes	Yes	22
May et al. (2019)	Concurrent multiple-probe design across stimulus sets	Concurrent multiple-probe design across stimulus sets	Yes, at two weeks following post-testing for each participant stimulus set	Yes	No	22
Petursdottir and Hafliđadóttir (2009)	MBL across participants with an embedded AATD	Pre- post-test probes with control conditions	No	Yes	No	19
Petursdottir et al. (2008)	MBL across stimulus sets	MBL design across stimulus sets	No	Yes	Yes	19
Wu et al. (2019)	MBL across participants with an embedded AATD	Pre- post-test probes with control conditions	No	Yes	Yes	22

*AATD*, adapted alternating treatments design, *MBL*, multiple baseline.

Based on criteria as recommended by Schlosser and Sigafoos (2007) and Council for Exceptional Children (2014)

### Meta-analysis

Visual analysis (available in the online supplemental materials) did not reveal consistent differences in acquisition curves. In other words, some participants acquired foreign tacts faster than other relations, but not all. Table 3 shows the mean acquisition rates (SARs) for the studies in the meta-analysis. Foreign tact training produced the lowest SAR within just one of the seven studies (Dounavi, 2011)—most participants in this study acquired trained foreign tacts faster than FNI responses. On the other hand, FTT produced the highest SAR in two studies (Cortez et al., 2020; Dounavi, 2014), which meant that participants generally acquired foreign tacts slower than the listener, FNI, or NFI relations. The SAR for FTT was neither the lowest nor the highest in four studies (Cortez et al., 2021; Daly & Dounavi, 2020; Petursdottir & Hafliđadóttir, 2009; Wu et al., 2019). For example, all three Daly and Dounavi (2020) participants acquired the trained foreign tact relations in fewer trials than FNI relations. Still, only one participant acquired foreign tact relations in fewer trials than NFI relations. Wu et al.’s (2019) mand and FNI training conditions produced lower SARs than FTT; however, the SAR for FTT was superior to that of NFI training. Overall, mand training (18.8) produced the lowest average SAR, followed by FNI training (20.2), FTT (22.1), NFI training (22.6), and listener training (23.8).

**Table 3:** Average standard acquisition rate (SAR) (lower value is better) and post-test scores (higher value is better) for each training condition

Study		FTT (n = 23)	NFI (n = 13)	Listener (n = 12)	FNI (n = 13)	Mand (n = 4)
Cortez et al. (2020)	SAR Post-test	26.3 **98.6%**		**24.3** 55.6%		
Cortez et al. (2021)	SAR Post-test	**28** **97.9%**		**28** 72.9%		
Daly and Dounavi (2020)	SAR Post-test	13 **98.6%**	**10** 94.4%		15.7 70.0%	
Dounavi (2011)	SAR Post-test	**12.3** **99%**	17.5 81.7%		17 63.3%	
Dounavi (2014)	SAR Post-test	21.8 **99.3%**	18.5 96.7%		**17.5** 64.2%	
Petursdottir and Hafliđadóttir (2009) *	SAR Post-test	27.5 58.3% (70.8%*)	43.1 **58.3%** (69.4%*)	**14** **58.3%**	30 14.6% (37.5%*)	
Wu et al. (2019)	SAR Post-test	24 **85.3%**	26.3 62.1%		21.8 54.7%	**18.8** 82.4%
Overall average SAR Overall average post-test score	Mean (SD) Mean (SD)	22.1 (9.8) **93.6%** (16.3), 93.7%*	22.6 (14.7) 79.0% (31.1), 79.6%*	23.8 (12.0) 61.5 (21.7)	20.2 (8.2) 55.8% (27.5), 57.4%*	**18.8** (6.2) 82.4% (26.2)

*FTT*, foreign tact training, *NFI*, native-to-foreign intraverbal, *FNI*, foreign-to-native intraverbal.

*Denotes scores with emergent listener post-tests included in the dataset

Foreign tact training achieved the highest mean post-test scores in all seven studies (Table 3). The within-subjects tests (Table 4) revealed participants’ mean FTT post-test scores were significantly higher than NFI, FNI, and listener training. FTT produced slightly higher mean scores than mand training, but the difference was not statistically significant. However, Wu et al. (2019) conducted mand training with the item to be requested in view of the participant, meaning it was a combination of foreign mand and tact relations under convergent multiple control (Michael et al., 2011). The Mann–Whitney U independent samples t-test indicated no significant differences in mean FTT post-test scores for children (*Mdn* = 100) and adult participants (*Mdn =* 100), U = 417, *p* = .203.

**Table 4:** Within-subjects comparisons between post-test scores for foreign tact training (FTT) and all other conditions

Training condition comparisons	n	Mean difference	Wilcoxon W statistic	*p* value	Effect size (rank biserial correlation)
FTT > NFI	13	8.9%	81	*0.007	0.780
FTT > Listener	12	29.5%	75	*0.003	0.923
FTT > FNI	13	35.2%	78	*0.001	1.00
FTT > Mand	4	3.71%	4	0.395	0.333

*NFI*, native-to-foreign intraverbal, *FNI,* foreign-to-native intraverbal.

*Denotes a statistically significant result

Foreign tact training produced the highest average EIS (5.1), followed by NFI training (4.9), mand training (4.7), listener training (3.3), and FNI training (3.1). Statistical analysis revealed no significant differences between FTT and the other training conditions—except FNI training (W = 77, *p* = .002).

## Discussion

This review adds to the growing literature on emergent foreign language learning. We found FTT produced high levels of emergent verbal relations for most participants. An explanation for the emergence of untaught NFI relations is that they share common stimulus and response topographies (covert native word and overt foreign word) with trained foreign tact relations (Petursdottir et al., 2008). According to naming theory, FTT stimuli are likely to evoke covert native responses and overt foreign vocal responses in verbally competent learners. It is difficult to determine if this occurred, as covert vocalizations are private events. Also, no authors reported learners’ overt native tacts during FTT.

**Fig. 2:**
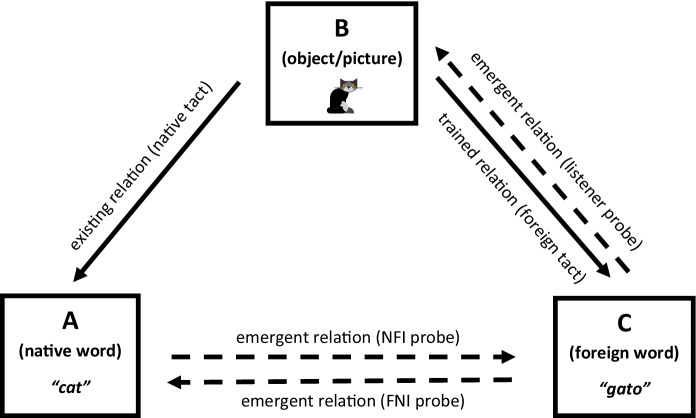
Existing, trained, and emergent relations following foreign tact training. *Note. NFI,* native-to-foreign intraverbal; *FNI,* foreign-to-native intraverbal

An alternative explanation (Fig. 2) is FTT learners derived equivalence relations between the native word, the object/picture, and the foreign word (Daly & Dounavi, 2020; May et al., 2013). Stimulus equivalence theory states that when stimulus A (native word) is related to B (object/picture), and B (object/picture) to C (foreign word), several relations may emerge without further training (Sidman, 2018). In all but one study (Cortez et al., 2020), experimenters ensured that participants could tact each target in their language either before (Cortez et al., 2021; Daly & Dounavi, 2020; Dounavi, 2011, 2014; Matter et al., 2020; Petursdottir & Hafliđadóttir, 2009; Petursdottir et al., 2008; Wu et al., 2019) or during training (May et al., 2016). Therefore, participants could relate stimulus B (object/picture) to A (native word) and B (object/picture) to C (foreign word) following FTT. Experimenters then tested participants’ emergent responses, demonstrating a range of equivalence relations: NFI probes tested for the emergence of untrained A–C equivalence relations; FNI probes tested C–A, and listener probes C–B. Then, the contextual cues that likely occasioned participants’ derived equivalence responses were the experimenters’ vocal stimuli—“What is the Spanish word for cat?,” “How do you say Gato in English?,” “Point to Gato,” “What do you call this in Spanish?” Pure mands, on the other hand, are evoked by motivating operations, not discriminative stimuli (Skinner, 1957)—the mands in Wu et al. (2019) were multiply controlled and probably tested B (object/picture) to C (foreign word) relations. However, the authors did not provide contextual cues consistently between FTT trials and mand post-tests, which may have caused the low levels of foreign manding following FTT. It is also possible that the tacts Wu et al. (2019) taught during FTT failed to emerge as mands because the tact training stimuli did not function as reinforcers (Wallace et al., 2006).

The meta-analysis compared emergent learning outcomes from FTT with outcomes from other verbal operant training procedures; FTT occasioned a significantly higher mean number of untrained verbal responses than intraverbal (FNI or NFI) or listener training and was more efficient than FNI training. Foreign tact training also produced a higher efficiency score (EIS) than NFI, mand, and listener training, but the differences were not statistically significant. Although results are preliminary due to the small number of studies, the aggregated data support the findings of several single-subject studies (e.g., Cortez et al., 2020, 2021; Daly & Dounavi, 2020; Dounavi, 2011). Furthermore, the findings suggest that teaching foreign language speaker skills is more efficient than teaching receptive skills, consistent with research on emergence in language programming. For example, Contreras et al. (2020) found tact or intraverbal training produced more emergent responses than listener training. In the present review, tact training was the most efficient condition; listener training and FNI were the least efficient conditions. Cortez et al. (2020) suggested that FTT is often effective at producing emergent foreign language responding because it provides opportunities to practice and reinforce the spoken foreign word.

We found no statistically significant difference in emergent responses between adults and children. Several researchers have previously posited a difference (e.g., Cortez et al., 2020; Daly & Dounavi, 2020; Dounavi, 2014; Petursdottir & Hafliđadóttir, 2009); however, our examination of aggregate data did not confirm this position. If differences exist, individual learning histories likely impact learners’ ability to derive emergent relations, as derived relations are learned behavior resulting from a history of multiple-exemplar instruction (Barnes-Holmes et al., 2018; Rehfeldt, 2011). Multiple-exemplar instruction may improve emergent learning outcomes by developing and reinforcing a repertoire of derived relations in less verbally competent learners for whom derived relations do not consistently or readily emerge (Lafrance & Tarbox, 2020). It is also likely that training arrangements, including mastery criteria, affected emergent outcomes.

Instructional mastery criteria varied across studies, and several experimenters (Cortez et al., 2020; Matter et al., 2020; May et al., 2019; Petursdottir & Hafliđadóttir, 2009) discontinued training phases before participants attained criterion-level responding, which may have affected emergent outcomes. Although not directly examined by the studies in this review, researchers have found that variability in training criteria can impact the emergence and maintenance of derived relations. For example, Fienup and Brodsky (2017) compared the levels of emergent learning resulting from two different training mastery criteria. Their results showed that more stringent training criteria produced higher levels of emergent responding. Similarly, the two studies in our meta-analysis with the lowest instructional mastery criteria (Petursdottir & Hafliđadóttir, 2009; Wu et al., 2019) produced the lowest average FTT post-test scores. These findings suggest that the production and retention of emergent relations depend on the strength of directly trained relations. In other words, it is the strength of participants’ trained skills that determine the strength and longevity of untrained skills (Critchfield & Twyman, 2014).

Lastly, the reviewed studies failed to examine response maintenance consistently. Maintenance of trained and untrained emergent responses are vital components of any emergent learning program (Wu et al., 2019), yet less than half of the studies reported any follow-up data (Cortez et al., 2021; Daly & Dounavi, 2020; Matter et al., 2020; May et al., 2019). Evaluating emergent outcomes requires a rigorous empirical assessment of learning maintenance over the long term.

This review has some limitations that the reader should consider. First, we excluded several studies that did not include standalone FTT conditions but did evaluate emergent foreign language learning outcomes (e.g., Cao & Greer, 2018; Haegele et al., 2011; May et al., 2016; Petursdottir et al., 2014; Polson & Parsons, 2000; Rosales et al., 2011, 2012) because we aimed to evaluate FTT’s outcomes, which required studies with at least one standalone FTT procedure to avoid the risk that combined procedures might produce confounding effects. Also, we chose not to search gray literature, which limited the number of included studies to peer-reviewed ones only. We consider our results preliminary data due to the small number of eligible studies. Second, we evaluated overall training efficiency based on the number of training trials conducted, not the duration of training, because no studies reported the total time required for each condition, and only two studies reported approximate session length (Cortez et al., 2021; Wu et al., 2019). A final limitation, common to any literature review, concerns the acknowledged bias within publications toward studies that produce positive findings (Torgerson, 2006). There is less potential for publication bias to negatively impact the results of the current meta-analysis, though, as we only included studies that directly compared at least two verbal operant training conditions. As such, studies showing negative FTT results would be just as likely to be published as studies showing positive results.

## Conclusion and Recommendations for Future Research

This review examined the effects of tact training on emergent foreign language learning outcomes. The key observation from these preliminary data was that FTT produced higher levels of emergent foreign language learning than other verbal operant procedures. This review raises several questions that warrant further research. First, why is FTT readily acquired for some learners but not all? Future research should consider what procedural variations might improve acquisition (e.g., number of stimuli; Kodak et al., 2019).

Second, why does FTT fail to produce emergence for some learners? It is possible that learners fail to emit emergent responses based on an insufficient reinforcement history of relational exemplars. Future FTT studies could include pre-assessment of learners’ relational responses and, if necessary, provide multiple-exemplar instruction before the commencement of the study. Pre-assessment of learners’ relational skills and selection of participants with similar pre-assessment results better controls for confounds associated with learner histories.

Third, how do FTT instructional mastery criteria affect emergence? Researchers should examine the preliminary finding that less stringent instructional mastery criteria negatively impacted emergent outcomes by using a within-subjects experimental design (e.g., an adapted alternating treatments design with different criteria assigned to each condition and counterbalanced across participants).

Fourth, what are the long-term learning outcomes associated with FTT? Further, what variables impact maintenance, and how might FTT be combined with other instructional procedures to improve outcomes (e.g., precision teaching; Critchfield & Twyman, 2014)? Researchers should look beyond the accuracy-based mastery criteria commonly employed in these studies to other mastery measures such as those employed within precision teaching’s fluency-based free operant response and measurement systems (Johnson & Layng, 1996; Bucklin et al., 2000).

Finally, we recommend future FTT research target a broader range of languages and instructional settings. Although the literature within the field is small, it highlights the considerable potential benefits that behavior analysis offers for optimizing foreign language learning programs.

## Supplementary Information

ESM 1 (DOCX 1813 kb)

## Data Availability

The datasets generated during and/or analysed during the current study are available from the corresponding author on reasonable request.
