# Supplementary material for: A Systematic Review of Emergent Learning Outcomes Produced by Foreign language Tact Training
Source: Anal Verbal Behav. 2022 Jul 6;38(2):157–78. doi: 10.1007/s40616-022-00170-z (PMC9747999; doi:10.1007/s40616-022-00170-z)
Supplement: Supplementary file 1 — (DOCX 1813 kb) [file 40616_2022_170_MOESM1_ESM.docx]

**Supplementary Material**

The following figures are supplementary material for the paper by Wooderson, J.R., Bizo, L. A., & Young, K. A systematic review of emergent learning outcomes produced by foreign language tact training. *The Analysis of Verbal Behavior.*

**Figure 1**

*Acquisition Curves (Part A) Adapted from Cortez et al. (2020)*


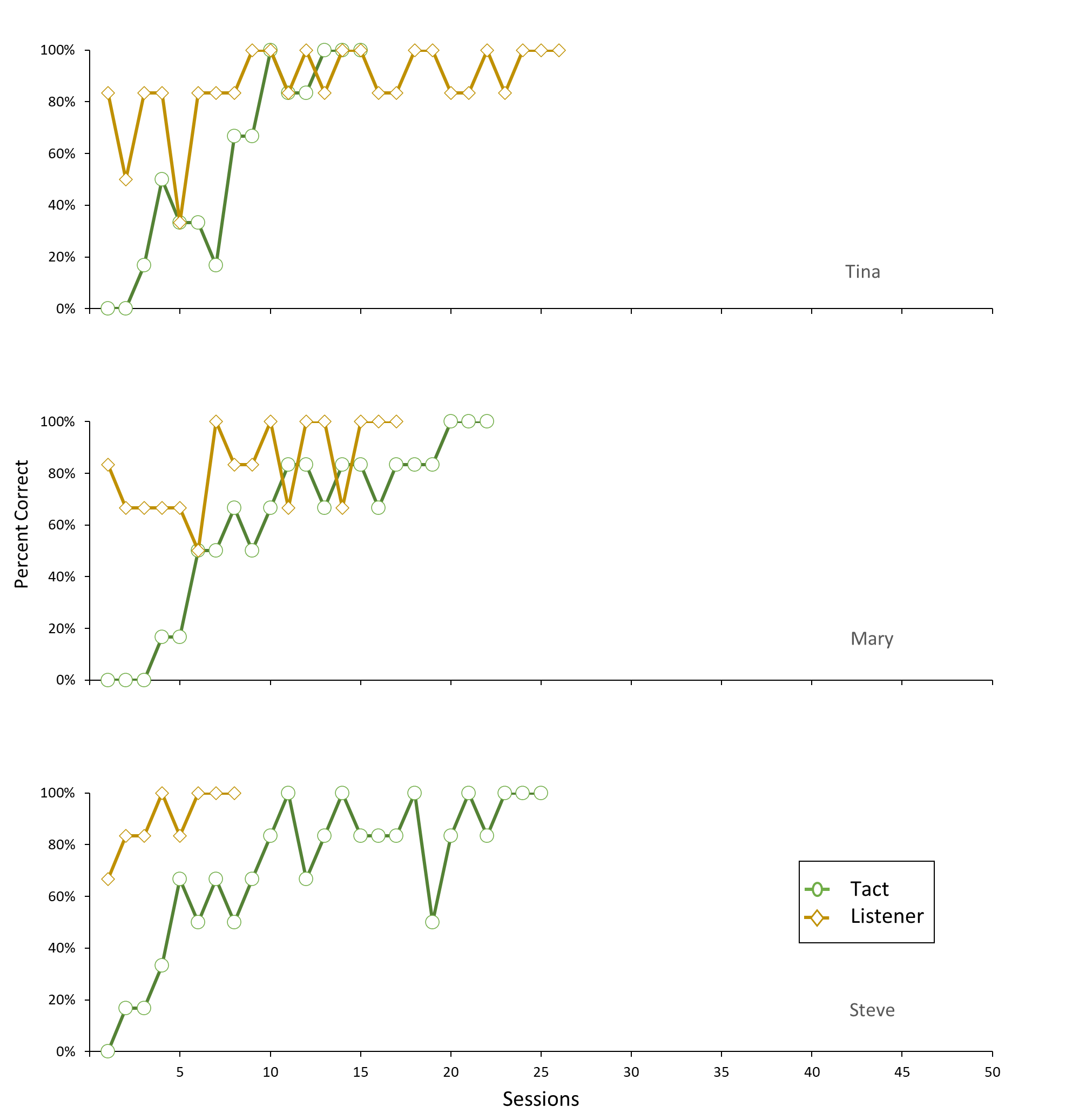


*Note.* This figure illustrates the differences in acquisition rates across tact and listener training conditions for Tina, Mary, and Steve in Cortez et al. (2020). The authors conducted training sessions in 6-trial blocks—one trial per target word. The mastery criterion was 100% correct responses in three consecutive trial blocks.

**Figure 2**

*Acquisition Curves (Part B) Adapted from Cortez et al. (2020)*


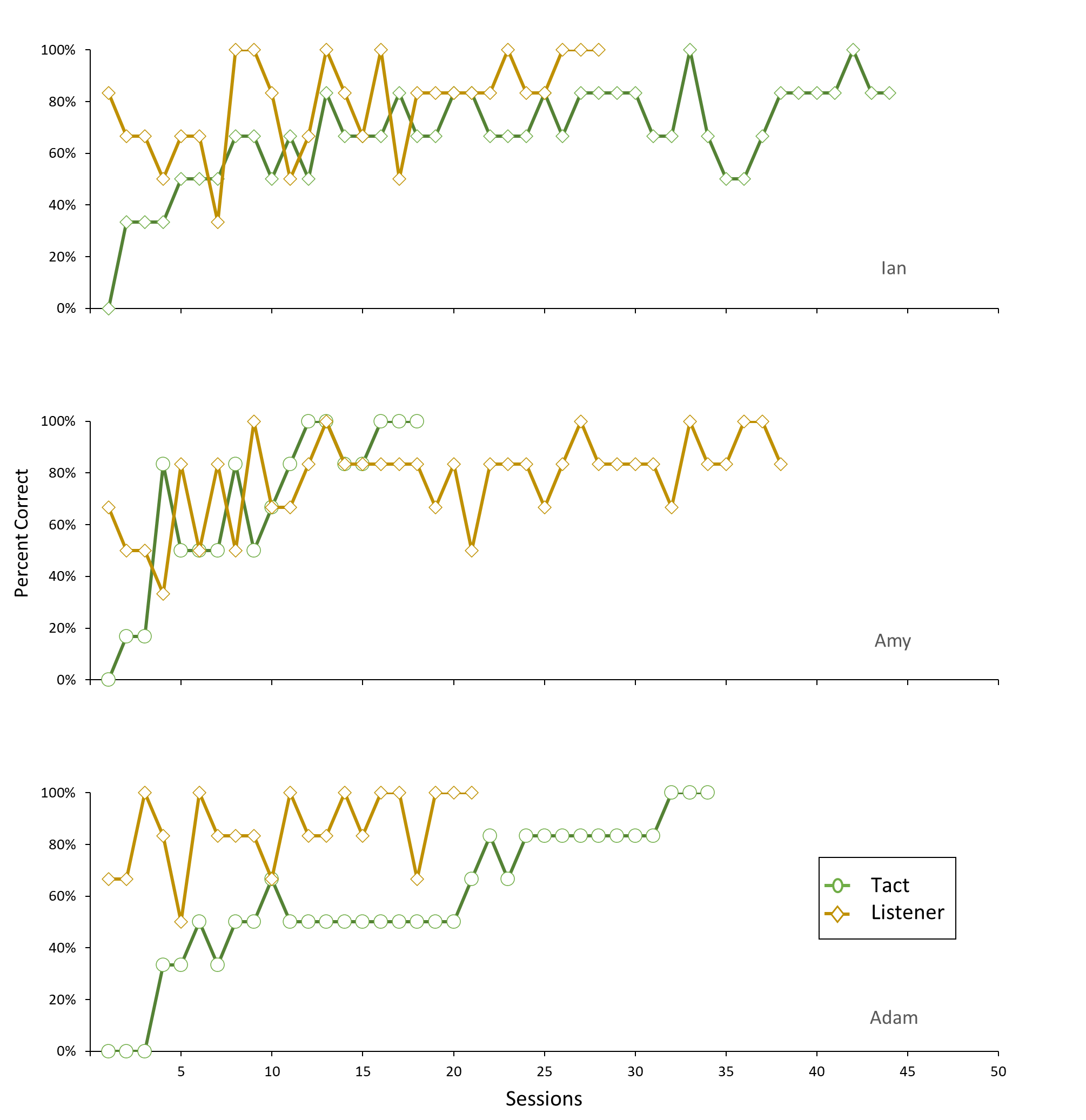
*Note.* This figure illustrates the differences in acquisition rates across tact and listener training conditions for Ian, Amy, and Adam in Cortez et al. (2020). The authors conducted training sessions in 6-trial blocks—one trial per target word. The mastery criterion was 100% correct responses in three consecutive trial blocks.

**Figure 3**

*Acquisition Curves Adapted from Cortez et al. (2021)*


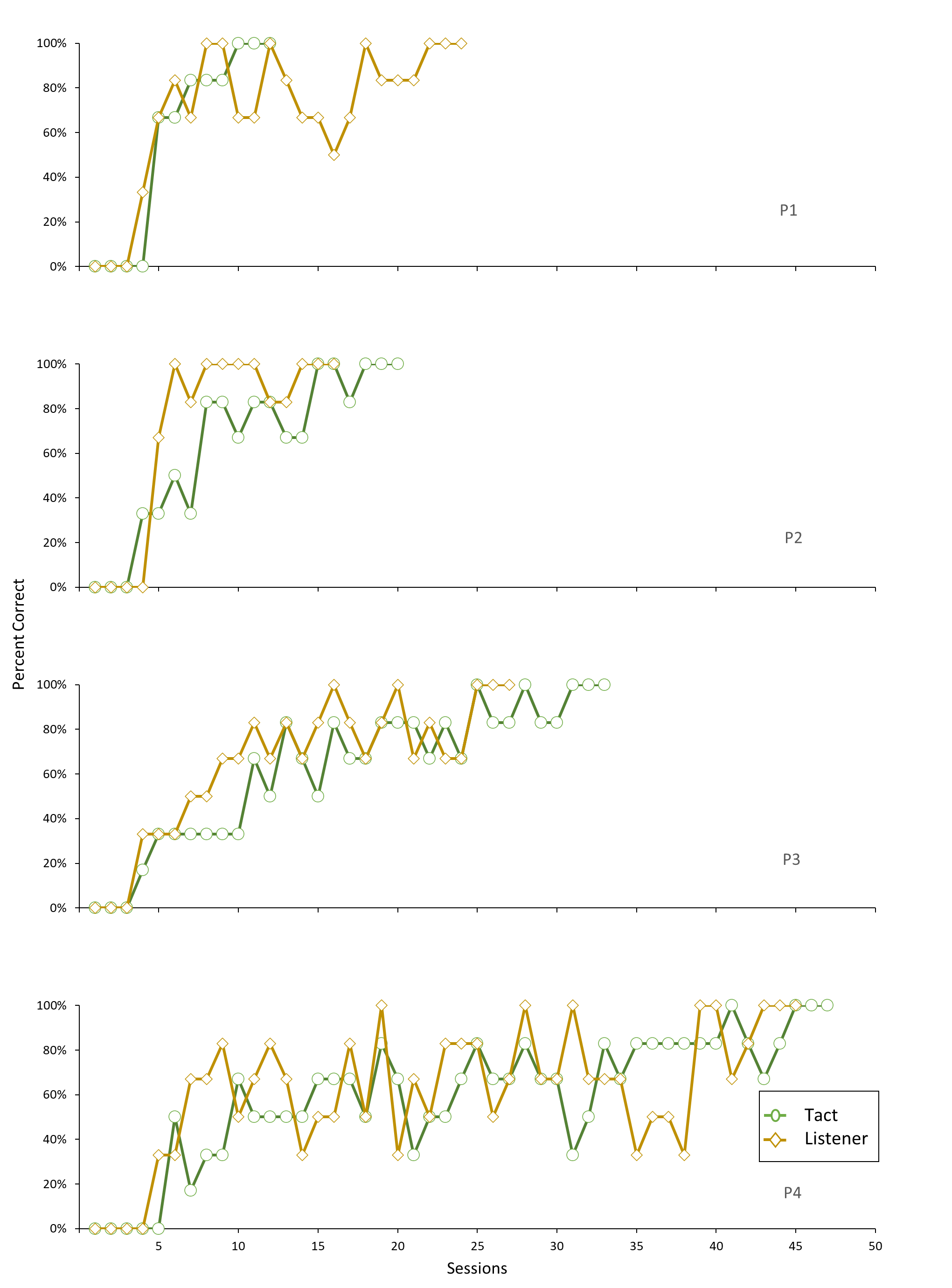
*Note.* This figure illustrates the differences in acquisition rates across tact and listener training conditions for Participants 1-4 in Cortez et al. (2021). The authors conducted training sessions in 6-trial blocks—one trial per target word. The mastery criterion was 100% correct responses in three consecutive trial blocks.

**Figure 4**

*Acquisition Curves Adapted from Dounavi (2011)*


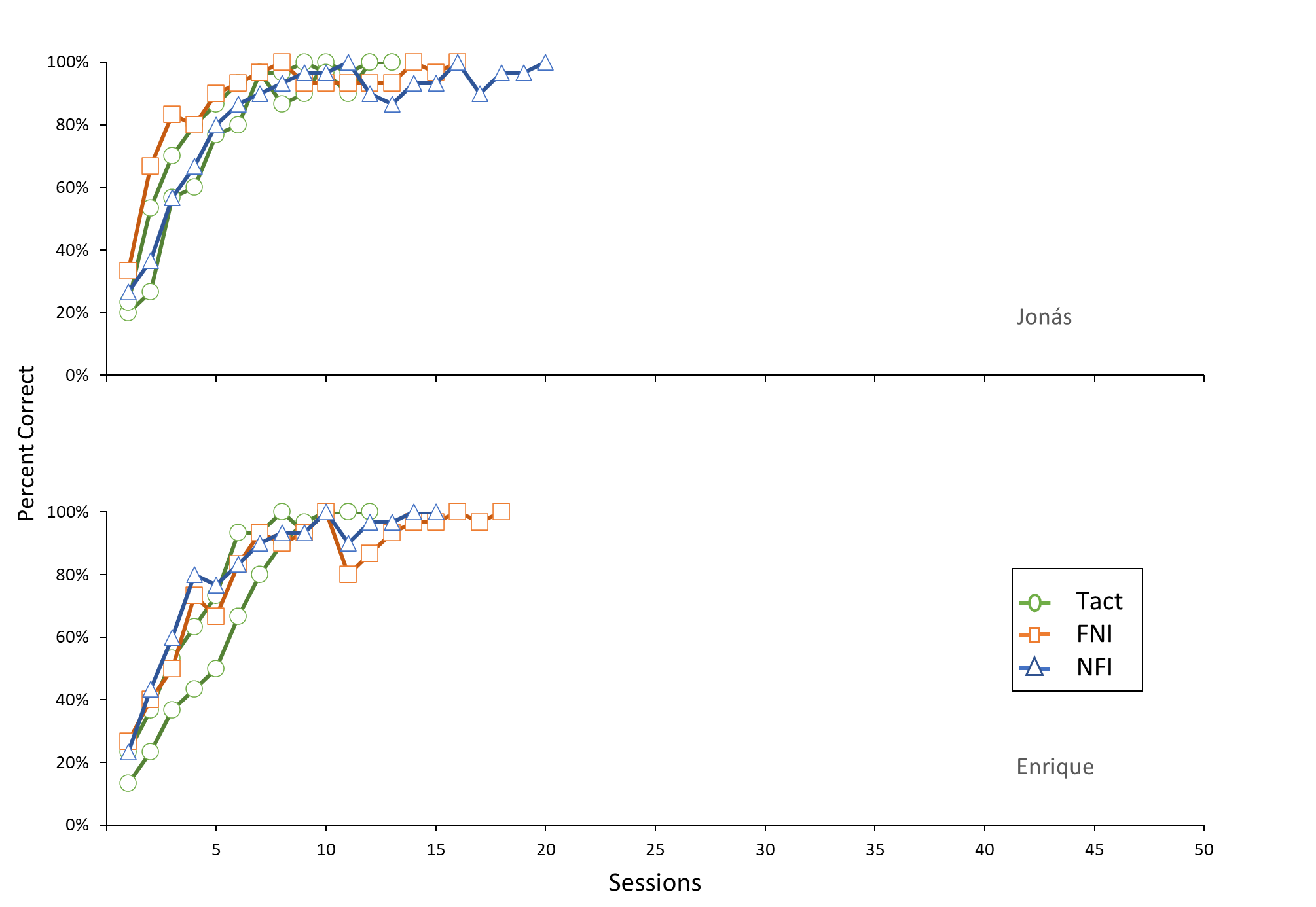


*Note.* This figure illustrates the differences in acquisition rates across tact, foreign-native intraverbal (FNI), and native-foreign intraverbal (NFI) training conditions for Jonás and Enrique in Dounavi (2011). The author conducted training sessions in 30-trial blocks—one trial per target word. The mastery criterion was 100% correct responses in two consecutive trial blocks.

**Figure 5**

*Acquisition Curves Adapted from Dounavi (2014)*


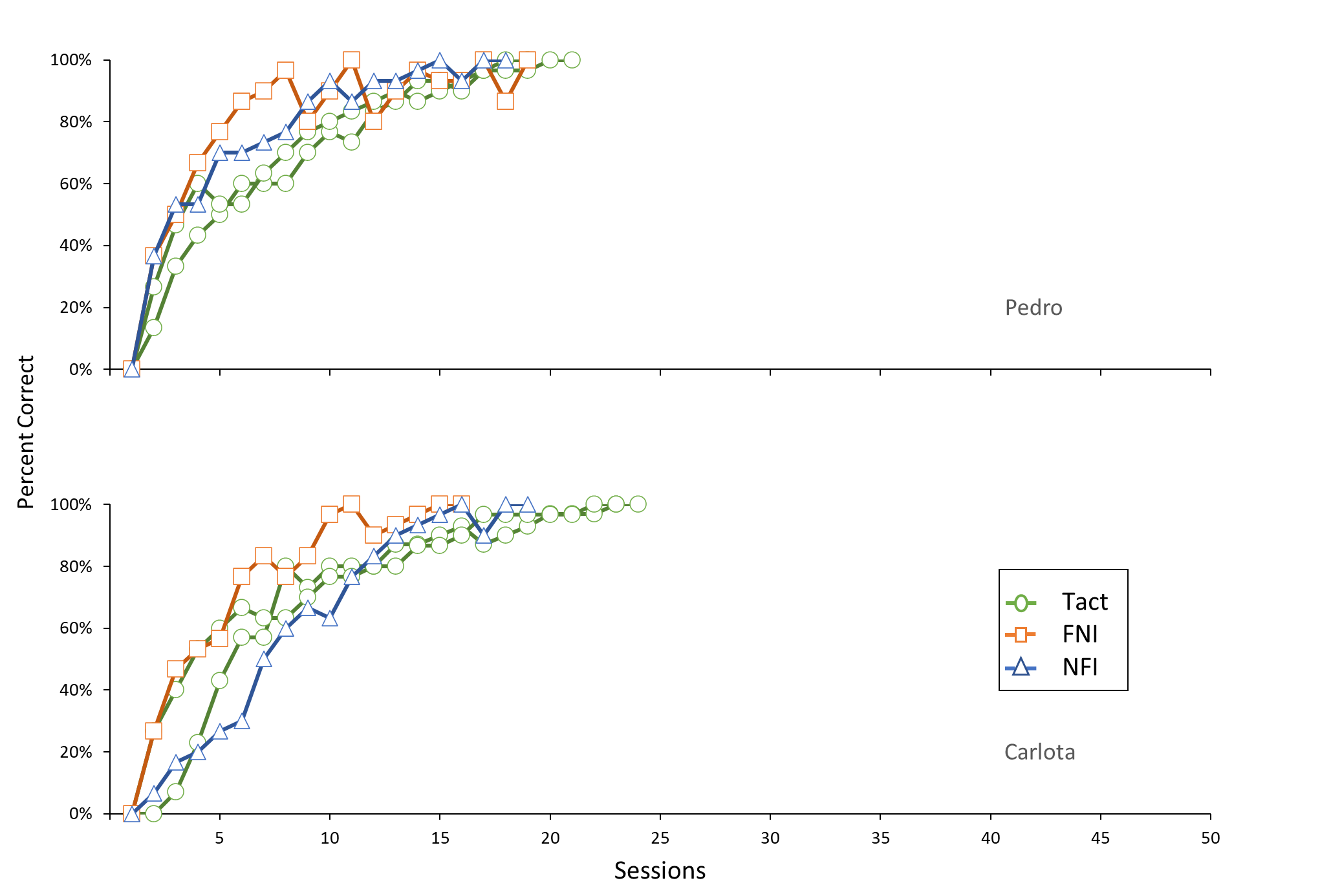


*Note.* This figure illustrates the differences in acquisition rates across tact, foreign-native intraverbal (FNI), and native-foreign intraverbal (NFI) training conditions for Pedro and Carlota in Dounavi (2014). The author conducted training sessions in 30-trial blocks—one trial per target word. The mastery criterion was 100% correct responses in two consecutive trial blocks.

**Figure 6**

*Acquisition Curves Adapted from Daly & Dounavi (2020)*


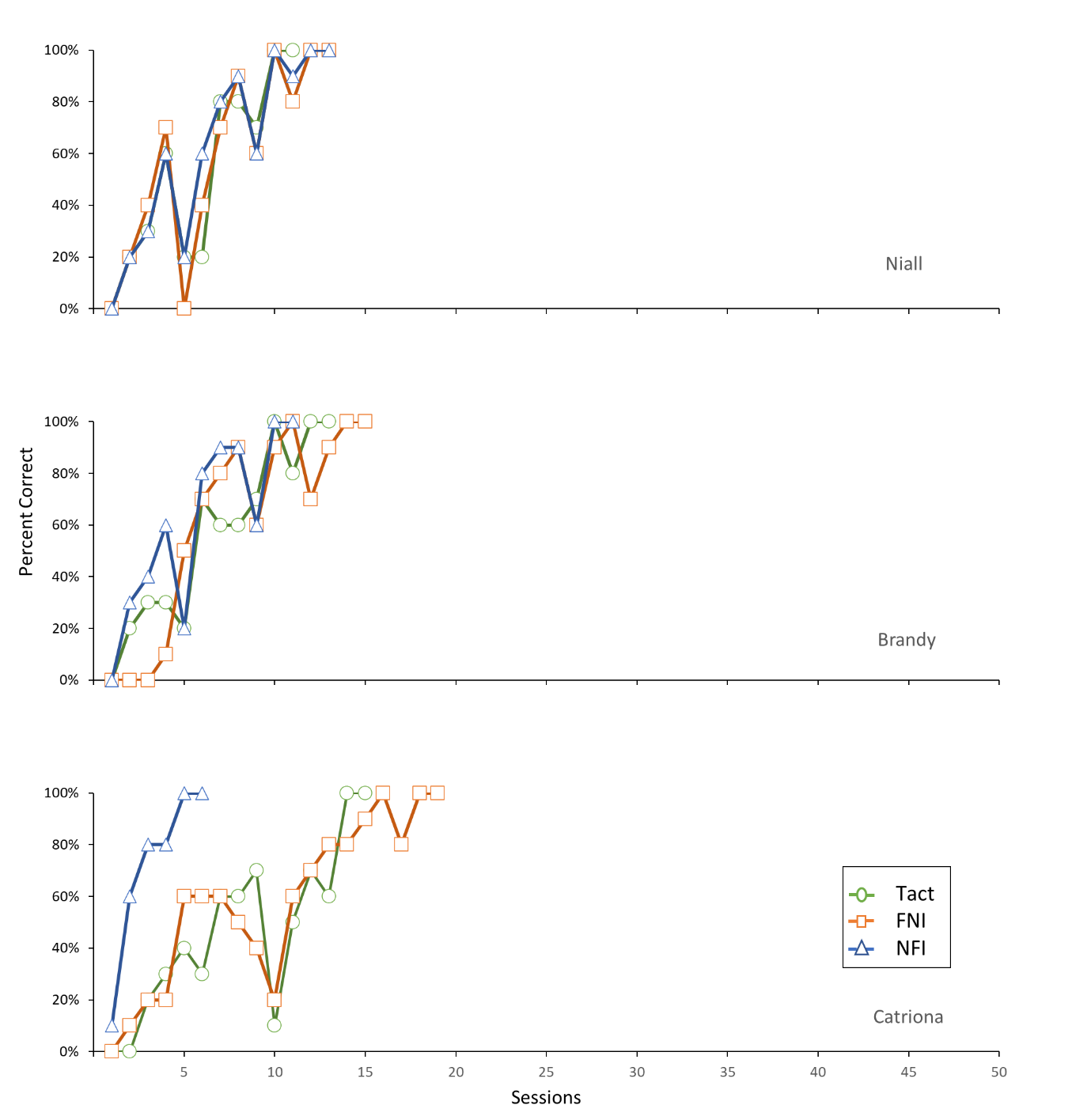


*Note.* This figure illustrates the differences in acquisition rates across tact, foreign-native intraverbal (FNI), and native-foreign intraverbal (NFI) training conditions for Niall, Brandy, and Catriona in Daly & Dounavi (2020). The authors conducted training sessions in 10-trial blocks—one trial per target word. The mastery criterion was 100% correct responses in two consecutive trial blocks.

**Figure 7**

*Acquisition Curves Adapted from Wu et al. (2019)*


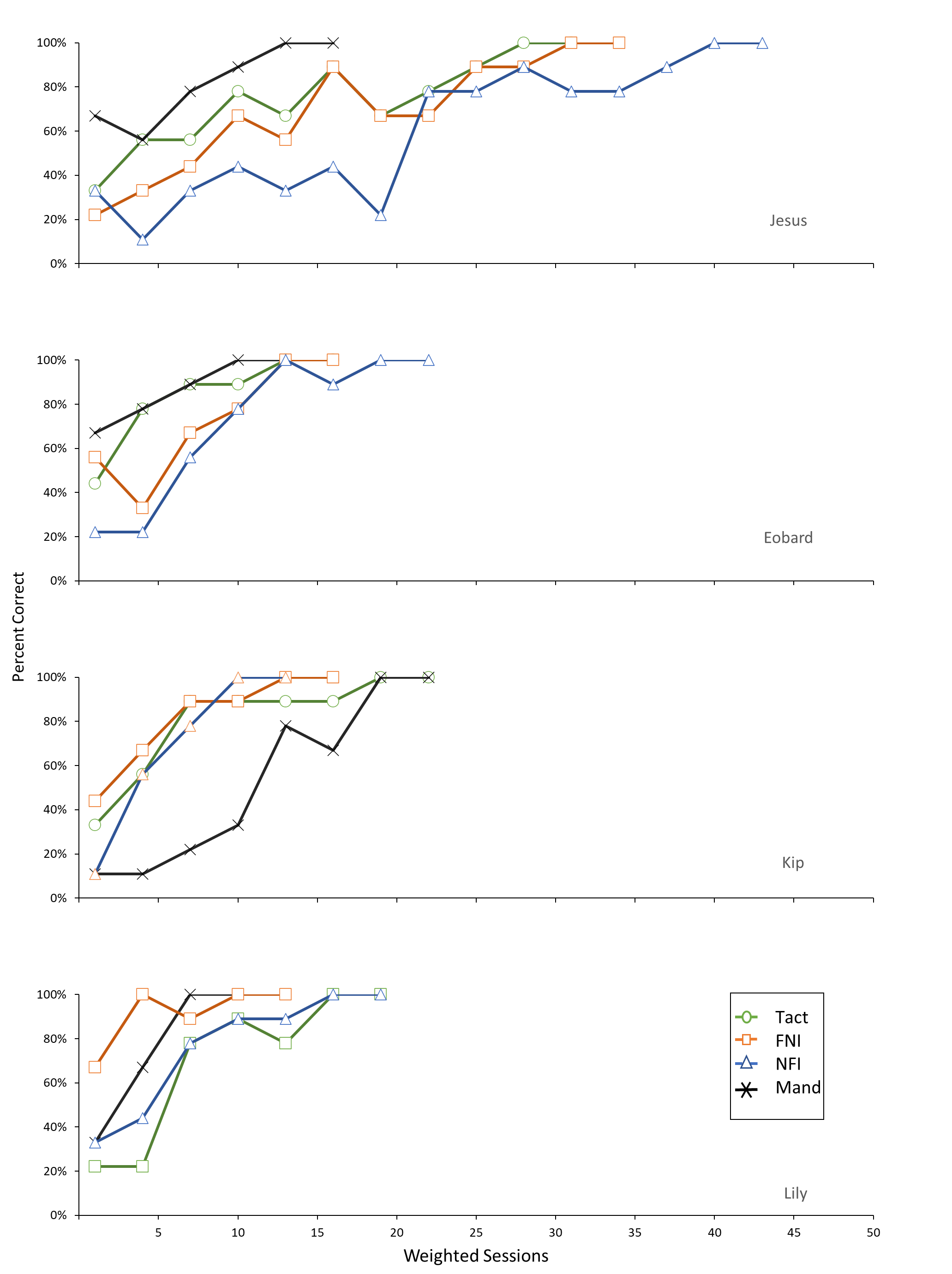
*Note.* This figure illustrates the differences in acquisition rates across tact, foreign-native intraverbal (FNI), native-foreign intraverbal (NFI), and mand training conditions for Jesus, Eobard, Kip, and Lily in Wu et al. (2019). The authors conducted training sessions in 9-trial blocks—three trials per target word. We weighted session counts by multiplying the number of sessions by three so that the data could be compared with the other studies in which each session comprised one trial per target word. The mastery criterion was 83.3% correct responses for two consecutive trial blocks.

**Figure 8**

*Acquisition Curves Adapted from Petursdottir & Hafliđadóttir, (2009)*


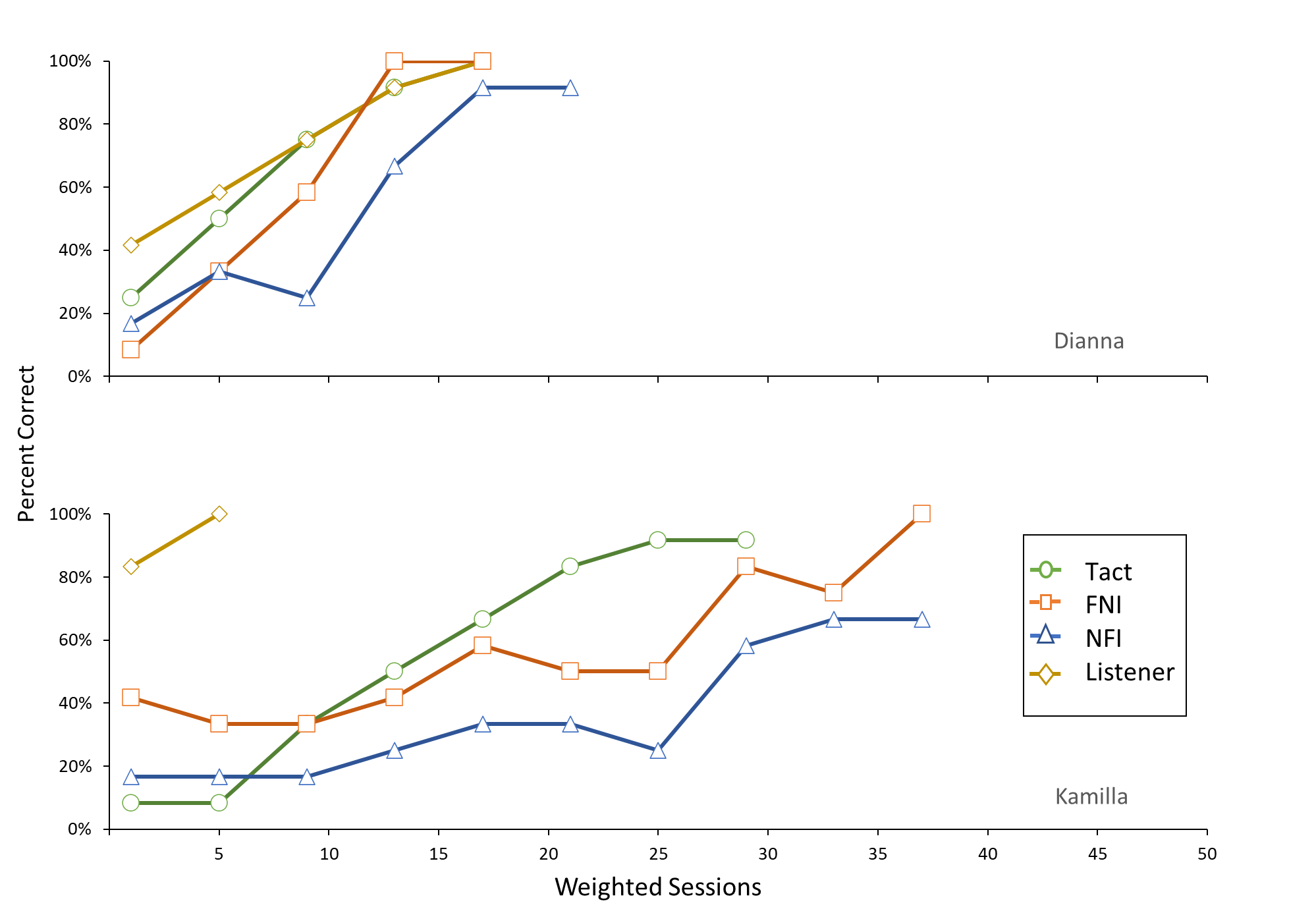
*Note.* This figure illustrates the differences in acquisition rates across tact, foreign-native intraverbal (FNI), native-foreign intraverbal (NFI), and listener training conditions for Dianna and Kamilla in Petursdottir & Hafliđadóttir (2009). The authors conducted training sessions in 48-trial blocks—four trials per target word. We weighted session counts by multiplying the number of sessions by four so that the data could be compared with the other studies in which each session comprised one trial per target word. The mastery criterion was 83.3% correct responses for two consecutive trial blocks.

**Figure 9**

*Combined Acquisition Curves for Each Study*


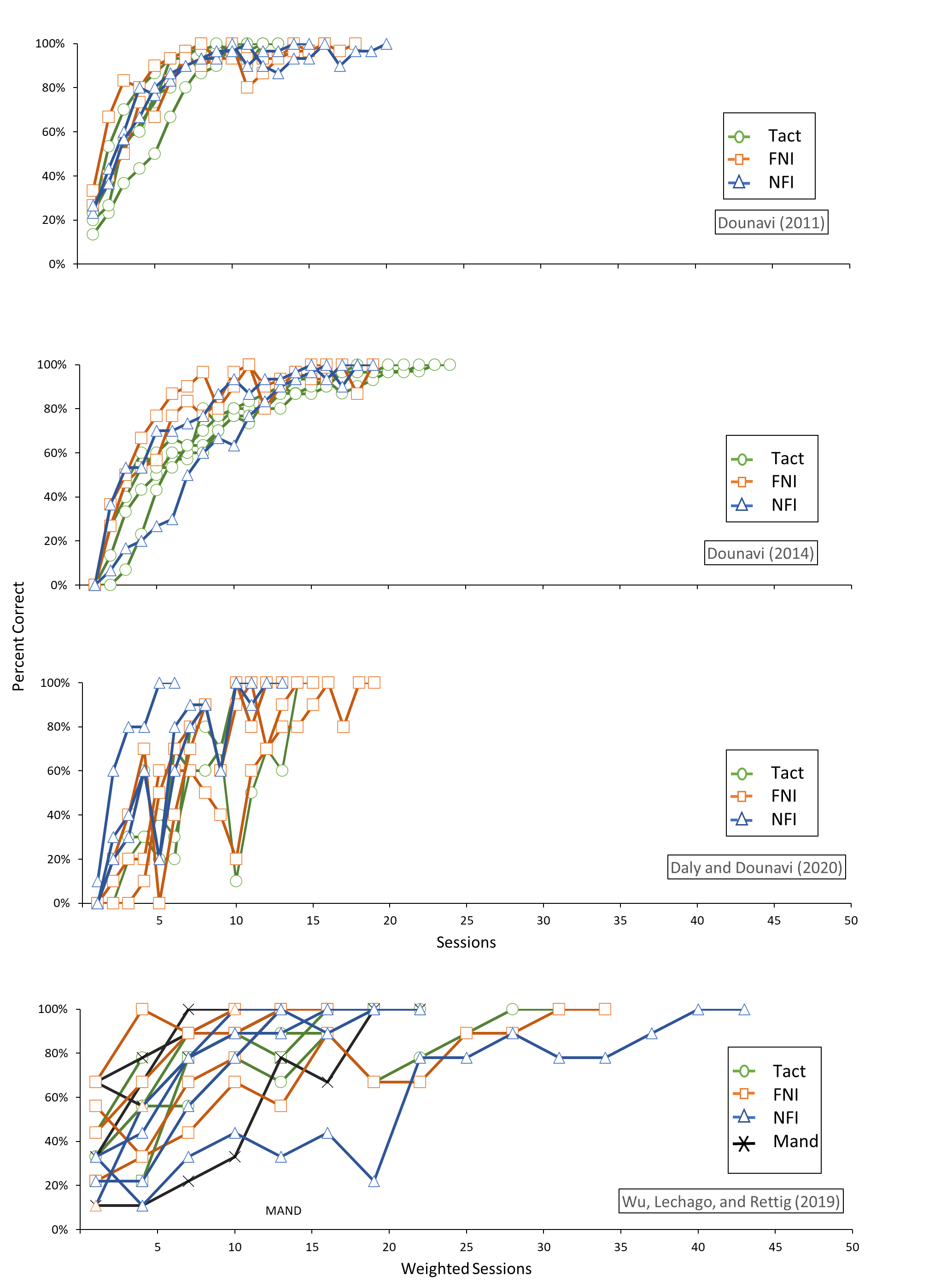


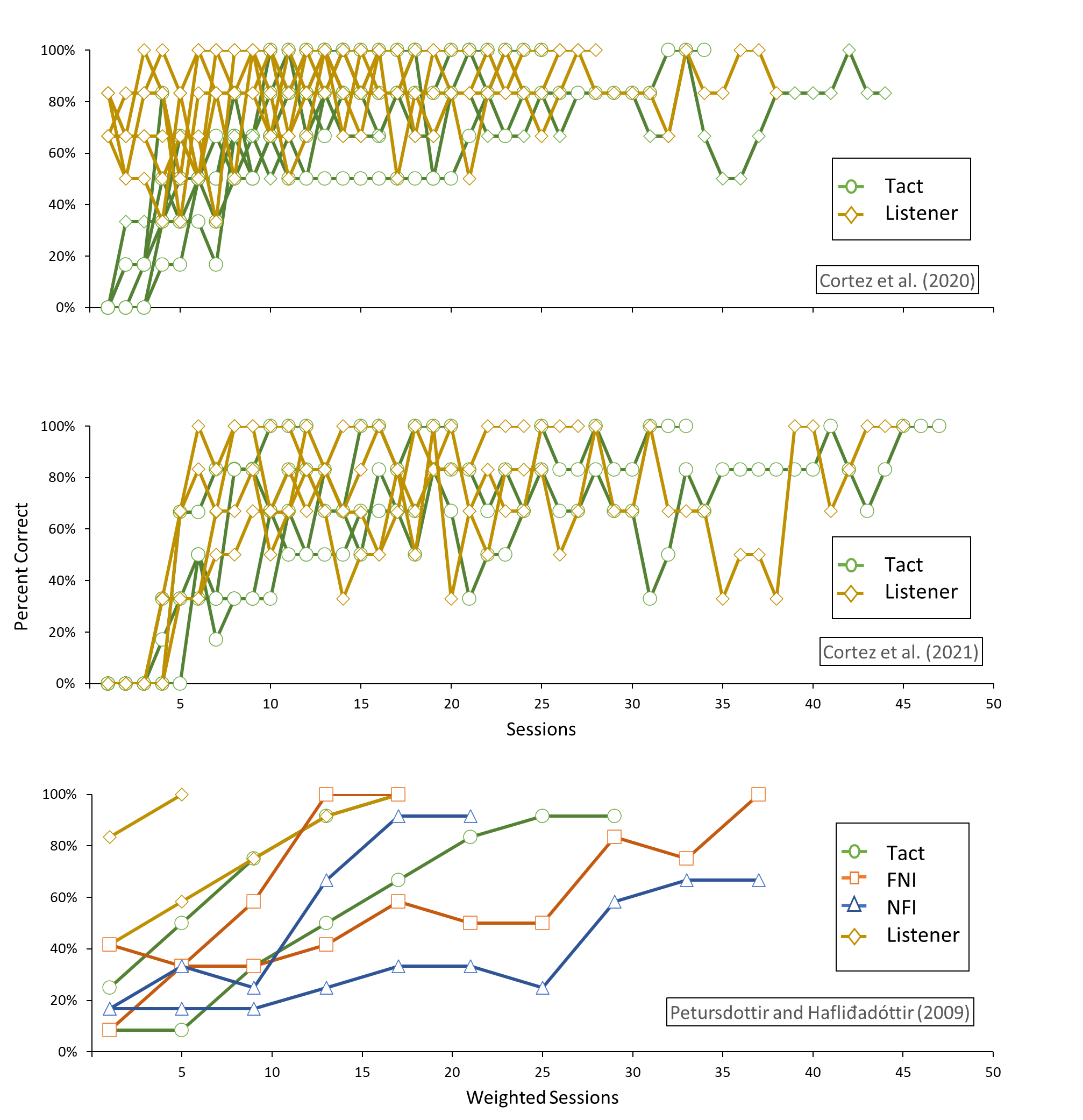


*Note.* This figure illustrates the differences in acquisition rates across tact, foreign-native intraverbal (FNI), native-foreign intraverbal (NFI), mand, and listener training conditions for all participants in each of the seven studies in the meta-analysis (Cortez et al., 2021; Cortez et al., 2020; Daly & Dounavi, 2020; Dounavi, 2011, 2014; Petursdottir & Hafliđadóttir, 2009; Wu et al., 2019).

# References

Cortez, M. D., da Silva, L. F., Cengher, M., Mazzoca, R. H., & Miguel, C. F. (2021). Teaching a small foreign language vocabulary to children using tact and listener instruction with a prompt delay. *Journal of Applied Behavior Analysis* (Advance online publication). https://doi.org/10.1002/jaba.885

Cortez, M. D., dos Santos, L., Quintal, A. E., Silveira, M. V., & Rose, J. C. (2020). Learning a foreign language: Effects of tact and listener instruction on the emergence of bidirectional intraverbals. *Journal of Applied Behavior Analysis, 53*(1), 484-492. https://doi.org/10.1002/jaba.559

Daly, D., & Dounavi, K. (2020). A comparison of tact training and bidirectional intraverbal training in teaching a foreign language: A refined replication. *The Psychological Record, 70*(2), 243-255. https://doi.org/10.1007/s40732-020-00396-0

Dounavi, A. (2011). A comparison between tact and intraverbal training in the acquisition of a foreign language. *European Journal of Behavior Analysis, 12*(1), 239-248. https://doi.org/10.1080/15021149.2011.11434367

Dounavi, K. (2014). Tact training versus bidirectional intraverbal training in teaching a foreign language. *Journal of Applied Behavior Analysis, 47*(1), 165-170. https://doi.org/10.1002/jaba.86

Petursdottir, A. I., & Hafliđadóttir, R. D. (2009). A comparison of four strategies for teaching a small foreign‐language vocabulary. *Journal of Applied Behavior Analysis, 42*(3), 685-690. https://doi.org/10.1901/jaba.2009.42-685

Wu, W. L., Lechago, S. A., & Rettig, L. A. (2019). Comparing mand training and other instructional methods to teach a foreign language. *Journal of Applied Behavior Analysis, 52*(3), 652-666. https://doi.org/10.1002/jaba.564
